# Supplementary material for: Predicting visual function by interpreting a neuronal wiring diagram
Source: Nature. 2024 Oct 2;634(8032):113–23. doi: 10.1038/s41586-024-07953-5 (PMC11446822; doi:10.1038/s41586-024-07953-5)
Supplement: Supplementary file 6 — CRF and ERF predictions for individual TmY4 and TmY9 cells. Analogous to Supplementary Data 3, but for TmY target types. Shown are the top four monosynaptic pathways, the strongest pathway passing through each of the top ten intermediary types (ranking from Extended Data Fig. 7), and the trisynaptic pathway Tm1–TmY–Dm3–TmY (see the section entitled Prediction of spatial normalization). [file 41586_2024_7953_MOESM6_ESM.zip › DataS4/TmY9q/720575940629181639.pdf]

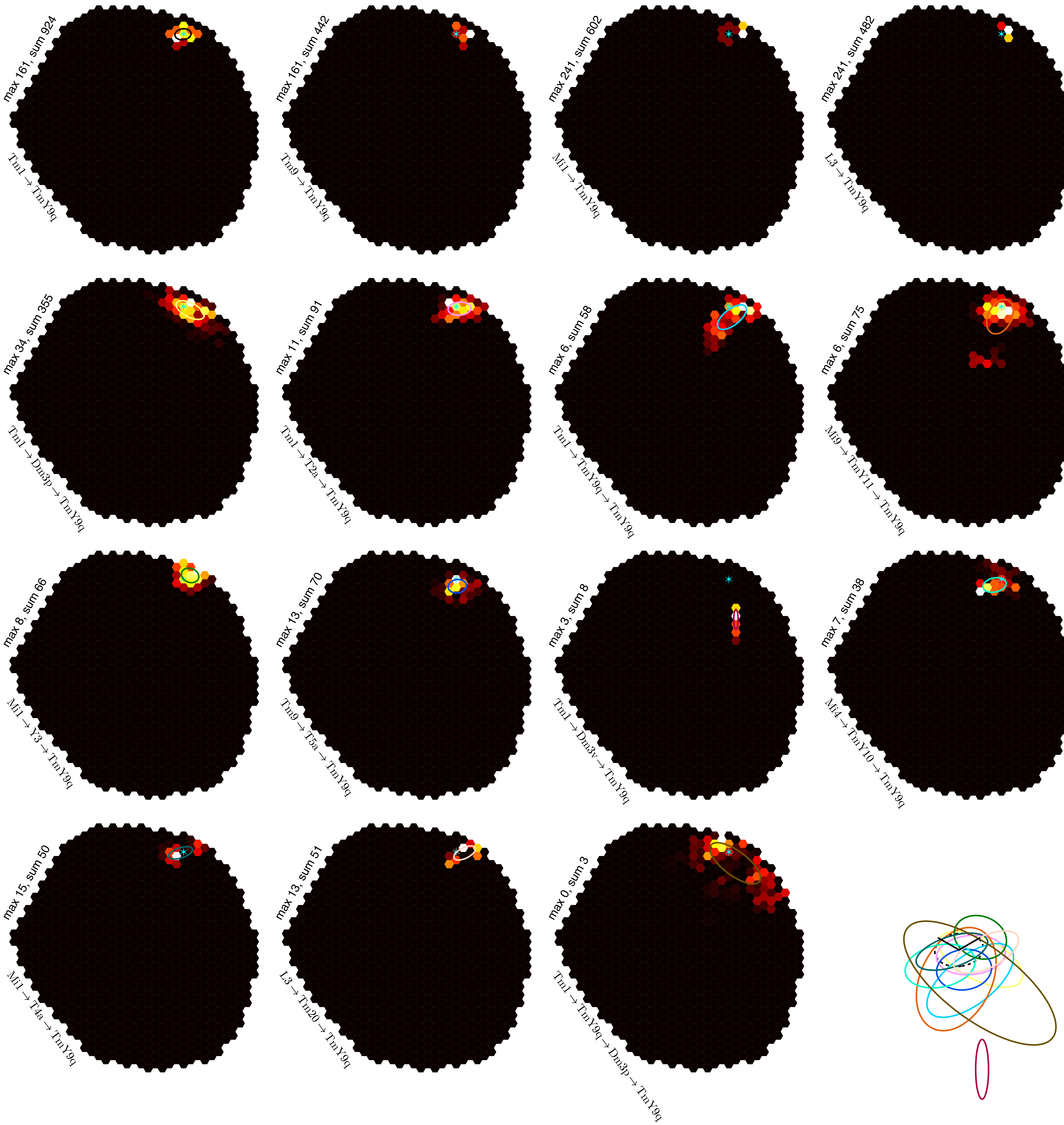
max 161, sum 924
Tm1 → TmY9q
max 161, sum 442
Tm9 → TmY9q
max 241, sum 602
Mi1 → TmY9q
max 241, sum 482
L3 → TmY9q
max 34, sum 355
Tm1 → Dm3p → TmY9q
max 11, sum 91
Tm1 → T2a → TmY9q
max 6, sum 58
Tm1 → TmY9q → TmY9q
max 6, sum 75
Mi9 → TmY11 → TmY9q
max 8, sum 66
Mi1 → Y3 → TmY9q
max 13, sum 70
Tm9 → T5a → TmY9q
max 3, sum 8
Tm1 → Dm3v → TmY9q
max 7, sum 38
Mi4 → TmY10 → TmY9q
max 15, sum 50
Mi1 → T4a → TmY9q
max 13, sum 51
L3 → Tm20 → TmY9q
max 0, sum 3
Tm1 → TmY9q → Dm3p → TmY9q
